# Green Synthesis of Superparamagnetic Iron Oxide Nanoparticles with *Eucalyptus globulus* Extract and Their Application in the Removal of Heavy Metals from Agricultural Soil

**DOI:** 10.3390/molecules27041367

**Published:** 2022-02-17

**Authors:** Karin Andrade-Zavaleta, Yessica Chacon-Laiza, David Asmat-Campos, Noemi Raquel-Checca

**Affiliations:** 1 Facultad de Ingeniería, Ingeniería Ambiental, Universidad Privada del Norte, Trujillo 13011, Peru; karin.andrade@outlook.com (K.A.-Z.); chaconlaizayessica@gmail.com (Y.C.-L.); 2 Dirección de Investigación, Innovación & Responsabilidad Social, Universidad Privada del Norte, Trujillo 13011, Peru; 3 Brazilian Center for Physics Research, Rio de Janeiro 22290-180, Brazil; nomifsc@gmail.com

**Keywords:** *Eucalyptus globulus*, green nanoparticle synthesis, chromium removal, cadmium removal, soil remediation

## Abstract

The green synthesis of metal oxide nanoparticles is presented as an excellent sustainable alternative for achieving nanostructures, with potential applications. This research provides important information regarding the influence of the type of solvent used in extracting organic reducing agents from *E. globulus* on the FeO NPs green synthesis protocol. A broad approach to characterization is presented, where UV-vis spectrophotometry suggests the presence of this type of nanoparticulate material. Likewise, the reduction mechanism was evaluated by FT-IR and the magnetic properties were evaluated by PPSM. In addition, characterizations were linked via elemental analysis (EDX), crystallographic characterization (XRD), electron microscopy (SEM/STEM), and Z potential to evaluate colloidal stability. The results show the influence of the type of solvent used for the extraction of organic reducing agents from *E. globulus*, and the effect on the synthesis of FeO NPs. In addition, the nanostructure material obtained showed excellent efficiency in the remediation of agricultural soil, eliminating metals such as Cr-VI, Cd, and, to a lesser extent, Pb.

## 1. Introduction

In recent years, advancement in nanotechnology research has grown exponentially. These increasingly important nanomaterials are made up of nanoparticles (NPs) smaller than 100 nanometers, giving rise to a wide field of applications in biomedicine [1,2,3,4,5], the food industry [6,7], environmental bioremediation [8,9,10,11], energy storage [12], aquaculture [13], and more. These increasing applications have generated a change of direction in research for the development of profitable and environmentally friendly technologies [14].

Consequently, the application of nanoparticles is proposed as an innovative solution for certain needs of society. The techniques for obtaining these NPs focus particularly on three methods: physical, chemical, and biological [15]. In the development of physical and chemical methods, limitations such as cost, low productivity, and high energy consumption are evident, as well as negative impacts on the environment and human health due to the use of solvents and surfactants, which are characterized as toxic, corrosive, and flammable chemicals [16,17,18]. Accordingly, the use of chemical methods for the synthesis of nanoparticles in biomedical applications was restricted, due to the chemicals’ toxicity, instability, and lower biocompatibility [19]. In addition, several methods and techniques have been linked to the reduction process of precursor salts using inorganic chemical agents [20], including the sol-gel method [21] and techniques such as hydrothermal synthesis [22], laser ablation [23], thermal annealing [24], and, recently, “electric explosion” [25]. These methods and techniques have achieved a good mastery of complete reduction synthesis processes; however, they are very complex, costly, and unsustainable (highly toxic).

The biological method, or green nanotechnology, is presented as an alternative that respects the ecosystem and allows the limitations of traditional methods to be overcome, thereby improving the production of nanomaterials through more efficient, sustainable, and environmentally friendly processes [26,27,28,29].

In the last decade, it has been shown that the green synthesis of nanoparticles uses different biological sources from algae [30,31,32,33,34], microorganisms such as fungi [3,35,36,37], and plants [38,39,40,41,42]. The wide application of th biological method is attributed to the fact that proteins, amino acids, organic acids, vitamins, and vegetable secondary metabolites, such as flavonoids, alkaloids, terpenoids, heterocyclic components, and polysaccharides, have an important role in the synthesis of metallic nanoparticles, acting as reducing agents and stoppers [15].

These reducing agents or metabolites vary, depending on the solvent with which the extract from the biological source has contact. The solvent has an important effect on the components of the extract; i.e., different mixtures of biomolecules are obtained using extraction solvents with slight differences in polarity [15]. Based on this observation, investigations have been carried out on the synthesis of NPs in various alcohols [15], including ethanol [15,43,44], due to its high potential to obtain the organic components essential for green synthesis.

*Eucalyptus globulus* is a species belonging to the Myrtaceae family [45]. The characterization of this organic material [46] has made it possible to find a diversity of polyphenolic constituents, flavonoids, and tannins; which have been reported in other works [47,48,49,50,51,52,53,54] as reducing agents for the synthesis of nanoparticles. This implies that this organic material can also act as a potential organic reducing agent. 

Various types of nanoparticles have been applied, with favorable results, in the remediation of soils that have been synthesized by a green route, as well as for the removal of inorganic pollutants [55,56,57], pesticide residues [58,59], and pharmaceutical contaminants [60].

The objective of this research is to provide important information related to the improvement of the process of the green synthesis of FeO NPs, specifically in relation to obtaining the extract of *E. globulus* as a reducing agent, with 96% alcohol and absolute ethanol solvents. Likewise, the application of nanostructures as a potential soil remediation agent, by eliminating metals such as Cr-VI, Cd, and, to a lesser extent, Pb, is considered.

## 2. Materials and Methods

The raw material of *Eucalyptus globulus* leaves, used in this research, came from the Huamachuco district, Sánchez Carrión province, La Libertad department, Peru. The soil sample was obtained from the district of Moche, department of La Libertad, Peru. Ultrapure water (Thermo Scientific, Barnstead Smart2Pure, Waltham, MA, USA) was used throughout the investigation. The extracts were prepared with the following solvents: alcohol 96% G.L. (Alkofarma Laboratory, Lima, Peru), and absolute ethanol (CAS No. 64-17-5—Merck Millipore, Darmstadt, Germany).

### 2.1. Preparation of the E. globulus Extract: Evaluation of the Type of Solvent

Fresh leaves of *E. globulus* were washed three times with ultrapure water to remove any type of impurities. Subsequently, a UNPA-MEMMERT model UM 55 plus a paraffin oven (Memmert GmbH Co. KG., Darmstadt, Germany) was used to dehydrate the prepared leaves at 70° C for 36 h and to eliminate the moisture present. After the established time, the burned leaves were removed and shredded.

Two samples were prepared separately with the solvents (alcohol 96% G.L. and absolute ethanol). For this process, 5 g of ground *E. globulus* were mixed with 50 mL of each solvent. Both prepared samples were placed under magnetic stirring (300 RPM) for around 30 min at room temperature (21 °C). Then, they were emptied into 15 mL falcon tubes and subjected to centrifugation at 3000 rpm (Hettich Zentrifugen, EBA 20C) for 15 min. The supernatant was separated using a diaphragm vacuum pump (GAST DOA-P704-AA). Finally, the extract obtained was covered with aluminum foil and stored at 4 °C for later use and analysis.

### 2.2. Sustainable Synthesis of Iron Oxide Nanoparticles (FeO NPs)

**Figure 1:**
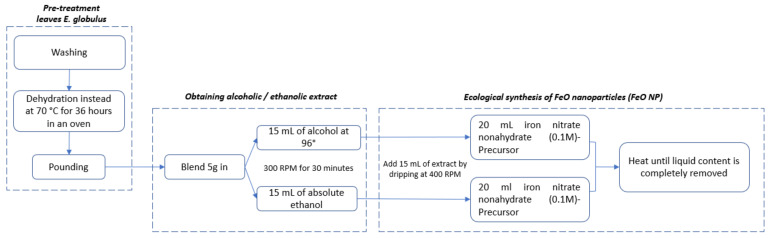
Scheme of the green synthesis protocol of iron oxide nanoparticles (FeO NPs), using extracts of *E. globulus*.

Figure 1 shows the scheme of the green synthesis protocol used in this investigation. The precursor iron nitrate nonahydrate (Fe(NO_3_)_3_ 9H_2_O) was initiated at a concentration of 0.1 M, which was diluted using ultrapure water as the solvent. Two precursor samples of 50 mL each were prepared, to which 15 mL of each previously prepared extract were added dropwise. The samples were placed in magnetic stirring (400 rpm at 21 °C). Finally, the liquid was evaporated using a water bath, until a black sediment was obtained, which indicated the presence of FeO NPs. The nanoparticles obtained were washed with ultrapure water and vortexed for 10 min to homogenize the sample, and then at ultracentrifuge (7000 rpm) for 15 min. This process was repeated three times (see Figure S2).

### 2.3. Characterization of FeO NPs

Once the nanoparticulate material was obtained, the first analysis was via UV-vis spectrophotometry (Hewlett Packard, 8452, Palo Alto, CA, USA). The equipment was calibrated in the range of 300 to 900 nm. This analysis aimed to find the peak surface plasmon resonance (SPR) of the material under study and to evaluate the stability over time of both samples, analyzing aliquots in periods ranging from 1 to 39 days. Likewise, FeO NPs were analyzed by Fourier Transform Infrared Spectrophotometry (FT-IR) (Nicolet iS50, Thermo Fischer Scientific, Maryland, USA) to evaluate the presence of some functional groups and thereby to consider the possible reduction mechanism present in the green synthesis. Morphological analysis was also performed, using a 200 kV/130 μA transmission electron microscope (TEM) (JEOL 2100F, Tokyo, Japan) equipped with a CCD camera (one view) in three modes: high resolution (HRTEM), scanning (STEM with a dark field annular detector, ADF), and, for elemental analysis, energy scattering X-ray spectroscopy (EDS, STEM-DF mode, and the Oxford energy spectrometer, Xplore). The samples for TEM analysis were prepared by placing the nanoparticulate material with acetone in ultrasound for about 30 min. This solution was dropped on the TEM grids, covered with carbon. Structural analysis was performed by X-ray diffraction (XRD) (empyrean diffractometer, Panalytical) with Cu-Kα radiation (λ = 1.54056 Å) at 45 kV and 40 mA. The information was obtained in the range of 20° < 2θ < 80° in Bragg Brentano geometry, spinner mode, with a step size of 0.026°. The stability analysis was complemented using characterization by Zeta potential from its electrophoretic mobility (Zeta plus—Zeta potential analyzer, Broookhaven Instrument Corporation, Holtsville, NY, USA). The results were reported as an average of ten different measurements and their standard deviation. Due to the nature of the nanoparticulate material (FeO), it was essential to characterize the magnetic properties using a DynaCool from the Quantum Design Physical Properties Measurement System (PPMS) of the Brazilian Center for Physics Research. Likewise, magnetization was evaluated as a function of temperature, which was carried out under zero field cooling conditions (ZFC) and field cooling (FC) (200 Oe probe), with hysteresis loops in the range of 5 K and 300 K, and field application up to 9 T.

### 2.4. Evaluation of the Elimination of Heavy Metals Present in Agricultural Soil

In the application stage, the influence of the FeO NPs colloid volume and its influence on the removal of heavy metals were evaluated. Samples of agricultural soil from the agricultural area of the Moche district, located in the province of Trujillo in Peru (an area currently affected by the presence of mining tailings in the water tributaries near the site) were used. Three similar samples were prepared by diluting 125 g of agricultural soil in 250 mL of ultrapure water, and maintaining magnetic stirring (600 rpm) for around 20 min/21 °C.

Regarding the colloid FeO NPs, three volumes were considered duly coded, as follows: sample M1 = 5 mL, sample M2 = 10 mL, and sample M3 = 15 mL. Each of these volumes was measured to a value of 50 mL with ultrapure water so that the colloidal samples were homogeneous in volume. Subsequently, each diluted agricultural soil sample was mixed with its respective volume of colloid FeO NPs, obtaining a total volume of 300 mL of sample, which was homogenized in magnetic stirring (250 rpm) for 30 min. A control sample (without FeO NPs colloid) of agricultural soil was prepared, diluted in 300 mL of ultrapure water, and homogenized in the same conditions.

For the quantification analysis of heavy metals (chromium, cadmium, and lead) by atomic absorption (Agilent Technologies, 200 series AA, Santa Clara, CA, USA), 5 mL of each of the samples were taken and 10 mL of HNO_3_ + 3HCl solution were added, filling with ultrapure water to a volume of 50 mL. These samples were digested until the volume was reduced to a value of 10 mL for 50 min.

Finally, all the samples obtained, volumetric to 50 mL with ultrapure water, were duly filtered with a diaphragm vacuum pump to avoid the presence of impurities. The samples were then ready for reading and analysis.

## 3. Results

### 3.1. Characterization by UV-vis Spectrophotometry

FeO NPs usually present a characteristic to the naked eye that is linked to light brown and black colors, due to their excitation against the interaction with electromagnetic radiation and the effect on the surface plasmon resonance (SPR); likewise, they are directly linked in size and morphology.

**Figure 2:**
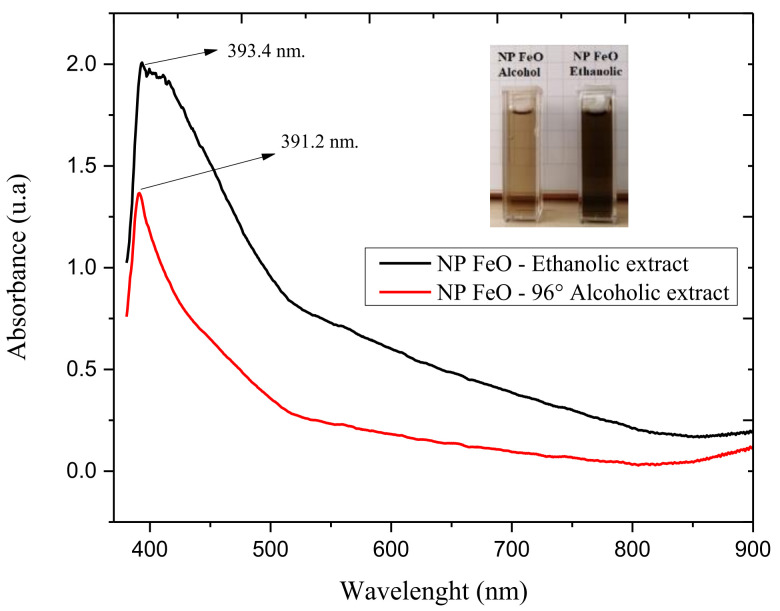
Results by UV-vis spectrophotometry of colloids of FeO NPs synthesized by the green route.

Organic extracts usually contain metabolites that act as potential reducers of metal salts. This reduction involves a process of formation of nanostructures. In this sense, as an initial characterization in a nanoparticle synthesis process, UV-vis spectrophotometric analysis was considered. Figure 2 provides the results of both colloids under evaluation, showing the presence of the SPR peak in typical ranges for this type of nanostructure (FeO NPs), specifically at 391.2 nm for the FeO NPs obtained using the *E. globulus* extract in the 96% alcohol solvent as the reducing agent, and at 393.4 nm using the extract in the absolute ethanol solvent. The difference between both spectra is related to the intensity of their absorbance. FeO NPs synthesized using the ethanol extract had more intensity, which is associated with the effect of absolute ethanol in extracting greater amounts of phenolic compounds. These compounds promote the reduction process of the precursor salt, generating a greater production of nanoparticles. Thus, it is understood that a higher intensity of the absorbance peak is synonymous with better production of nanoparticles. However, the final stabilization process and the plugging effect of the phenolic compounds are so excessive that a metal-phenolate complex with free charges is possibly generated, leading to aggregation processes and, therefore, instability over time. 

This effect does not occur with FeO NPs obtained using the 96% alcohol extract, where there is a balanced reduction process between the metal ions and the hydroxide ions of the extract. A successful plugging process may be considered, avoiding agglomerations and therefore generating stability over time. This is why the bandwidth is better than in the previous case. These procedures are demonstrated and corroborated by the other results described in this paper. 

**Figure 3:**
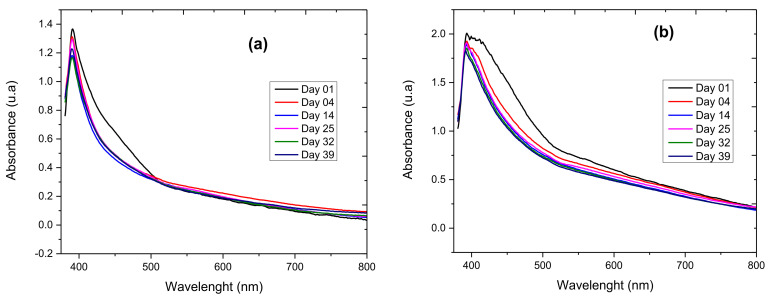
Spectrophotometric characterization for the colloidal stability analysis of FeO NPs with *E. globulus* extract, (**a**) using the 96% alcohol solvent and (**b**) using the absolute ethanol solvent.

The colloidal samples of the FeO NPs obtained were subjected to evaluation by spectrophotometric characterization to evaluate their stability over time. Figure 3a,b shows the behavior of the FeO NPs colloids obtained by green synthesis in both the 96% alcohol solvent and the absolute ethanol solvent during an interval from 1 to 39 days. In both cases, the dynamic behavior of the absorbance decreased as the days passed, indicating a possible agglomeration of the nanostructures. The bandwidth observed from inception for the sample with the ethanol solvent extract tended to decrease, which is linked to the formation of clusters of similar sizes; i.e., clusters with a tendency to be slightly monodisperse. Better stability and monodispersity behavior occurred when the 96% alcohol solvent is used.

Green synthesis procedures are being increasingly studied in recent years. However, it is important to consider that the concentration of polyphenols, or some type of functional group, defines the correct formation and, especially, the stability of the nanostructures. Chemically, there are reaction procedures of complexes of the metal salt (in this case, iron salt) and the process of Fe (III) reduction by oxidized polyphenols, which is suggested as a mechanism [61]. For this reason, the solvent used for the extraction of organic compounds is important, and this research contributes to that consideration for the first time.

### 3.2. Fourier Transform Infrared Spectroscopy (FT-IR)

**Figure 4:**
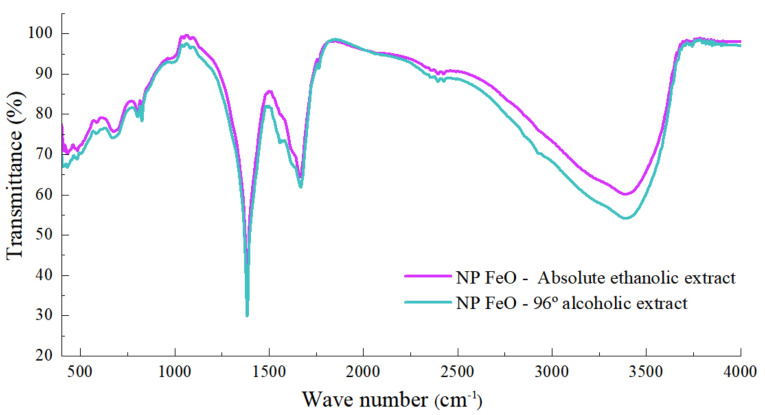
FT-IR characterization of FeO NPs colloids, mediated by *E. globulus* extract, using the 96% alcohol solvent and absolute ethanol solvent.

The FT-IR spectra of the leaves of *E. globulus* extracts in the 96% alcohol and the absolute ethanol solvents, and the respective FeO NPs, are shown in Figure 4. The peak is located at 1668 cm^−1^, corresponding to the aromatic ring C=N, 1382 cm^−1^ and the vibration -CN- of the amides, or the -CO- stretching of alcohols, carboxylic acids, and 1082 cm^−1^, corresponding to the C-O or C=O vibration. In addition, for the 96% alcohol solvent extract, peaks are located at 3400 cm^−1^ related to the H bond. 

Consequently, it could be concluded that the metabolites responsible for the reduction process are those that are related to the family of aromatic compounds.

It has been shown that the eucalyptus extract in both types of solvents presents simple phenols and derivatives of phenolic compounds, which could be responsible for the process of reduction of metal ions and, therefore, the formation of FeO NPs.

The reaction of the metal ion, iron nitrate nonahydrate, occurs with the presence of phenolic groups, as shown by the presence of aromatic groups (CH) from the extract of *E. globulus*. These include the monosubstituted benzene ring, the 1,4 disubstituted benzene ring, and the 1,2,3 trisubstituted benzene ring, attributable to the extract of *E. globulus*, which in turn contains phenolic components (3Ar-(OH)n) such as borneol, carvacrol, citronellal, etc. These components react with the metal ions (precursors), generating oxidation reduction and finally achieving stabilization, due to the presence of a greater amount of other hydroxide ions (OH) Equation (1).

(1) $${\mathrm{nFe}}^{+3}+3\mathrm{Ar}\;{\left(-\mathrm{OH}\right)}_{n}\;\to {\mathrm{nFe}}^{0}+3\mathrm{Ar}\left(=O\right)n+3{\mathrm{nH}}^{+}$$

The result of the reductive reaction of the precursor nanoparticle is an Fe^0^-phenolate complex by a chelating effect (substance-forming complexes with heavy metal ions), causing the nucleation and growth of the nanoparticles.

### 3.3. X-Ray Diffraction (XRD)

**Figure 5:**
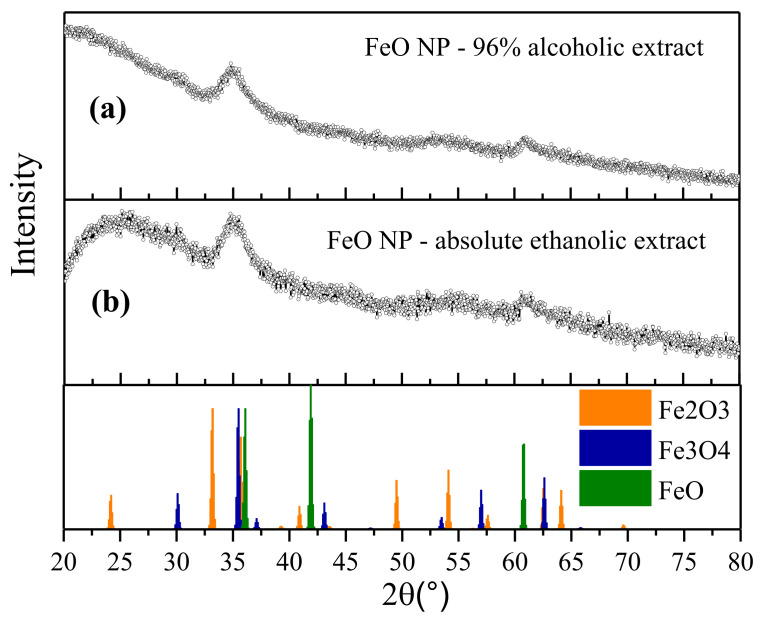
XRD analysis of FeO NPs synthesized with solvent extracts, (**a**) the 96% alcohol solvent, and (**b**) the absolute ethanol solvent.

Characterization by X-ray diffraction (XRD) was carried out. The results (Figure 5) show the presence of strong peaks at 35.6°, 54.2°, and 63.1°, related to the crystalline planes (311), (422), and (440), respectively, which are indexed to the magnetite magnetic phases (Fe_3_O_4_), with a slight contribution of the maghemite phase (γFe_2_O_3_).

For FeO NPs synthesized using an extract in the absolute ethanol solvent, a 2θ is shown at 25°, which can be attributable to the organic materials present in the extract and which acts as a stabilizing agent [61]. This peak was not observed for the other case under study. These results obtained are similar to those of other investigations, where a methodology for the green synthesis of FeO NPs was developed using other types of organic extracts. This similarity strengthens and sustains the conclusion that the synthesis was successful [17,62,63].

The information from the diffractograms obtained from FeO NPs was also used to determine the crystallite size. using the Debye-Scherrer equation. The results show measurements of 1.715 nm (FeO NPs using the absolute ethanol solvent extract) and 2.863 nm (using the 96% alcohol extract). The STEM results reinforce the sizes obtained with a range of similarity.

### 3.4. Elemental Composition—Energy Dispersive Spectroscopy (EDS)

**Figure 6:**
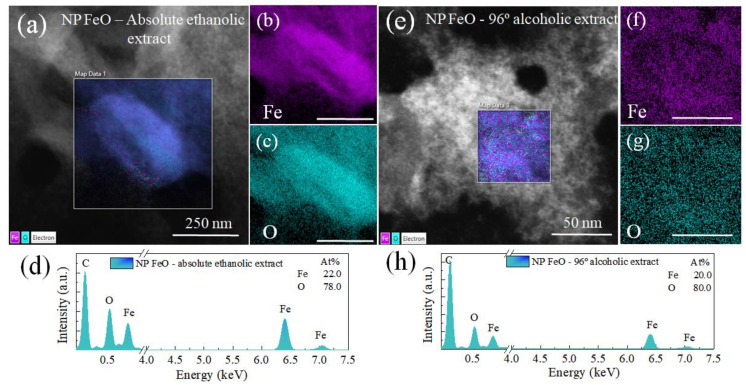
Result of characterization by EDX of FeO NPs mediated by green synthesis using extract in (**a**–**d**) the absolute ethanol solvent, and (**e**–**h**) the 96% alcohol solvent.

To determine the presence of the elemental composition of FeO NPs, an EDS characterization was performed. The results (Figure 6) show the presence of the elements iron and oxygen, without another type of element, confirming the quality and purity of the synthesis. The results show that the FeO NPs obtained with an extract in the absolute ethanol solvent have an elemental composition of 78.3% oxygen and 21.7% iron.

In the sample obtained with an extract in the 96% alcohol solvent, the values of O and Fe were very close to the previous case, with values of 80% and 20%, respectively. An important detail to highlight is that both samples did not show the presence of other types of elements, which is attributable to an efficient process of reduction and formation of iron in its oxide form Iron tends to generate reaction processes with air, generating the formation of layers of FeO [64].

In addition, an analysis of the electronic structure of the FeO NPs was carried out by means of EELS characterization. Figure S1 shows the peaks for the oxygen K edges and the Fe-L_2,3_ edges of both colloids under study. A difference in the intensity of peaks A and B is evident. Generally, a lower intensity is related to more oxygen vacancies in the nanomaterial, which allows us to deduce that the FeO NPs obtained with the extract in the absolute ethanol medium have more oxygen vacancies than the other study sample.

### 3.5. Characterization of FeO NPs by Transmission Electron Microscopy (TEM/STEM)

**Figure 7:**
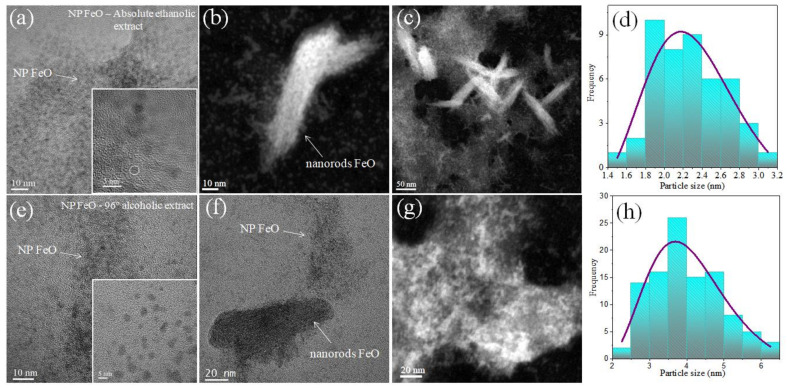
STEM characterization and size histograms of FeO NPs using extracts of *E. globulus* in solvent, (**a**–**d**) the absolute ethanol solvent and (**e**–**h**) the 96% alcohol solvent.

The STEM characterization confirmed the presence of nanoparticles with spherical morphology, and with sizes that are a function of the type of *E. globulus* extract used in this research. These observations confirmed the influence of the type of solvent. The FeO NPs obtained using an extract in the ethanol solvent (Figure 7a) demonstrated an average size of 2.34 ± 0.53 nm. Likewise, the presence of small agglomerates is very possibly linked to traces of the organic extract, which coincidentally relates to the peak of 2θ at 25° found in the XRD characterization, attributable to organic stabilizing agents, in addition to the organic radicals shown in the FT-IR.

For FeO NPs using the 96% alcohol solvent extract as a reducer (Figure 7e), the same type of geometry was evidenced, with an average size of 4.17 ± 1.22 nm, without showing evidence of the presence of any type of organic trace. The results corroborate those already obtained by calculating the crystallite size using the Debye-Scherrer equation by XRD.

### 3.6. Characterization of Magnetic Properties of SPIONs

**Figure 8:**
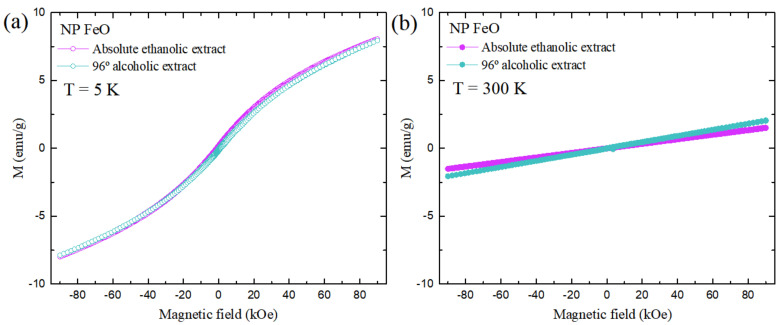
Saturation magnetization values as a function of field hysteresis applied to FeO NPs synthesized by the green path at (**a**) 5 K and (**b**) 300 K.

The characterization of the magnetic properties of the nanostructures under study (Figure 8) showed a response attributable to superparamagnetic materials (SPIONs), which in turn had a very similar response when they were at 5 K with an Ms magnetization value of around 7.94 emu/g. However, a decrease in this value was evidenced when the temperature increased to 300 K (1.501 emu/g for FeO NPs using extract in the absolute ethanol solvent, and 2.059 emu/g in the 96% alcohol solvent). This tendency of magnetization to decrease was related to the decrease in the size of the nanoparticle, as corroborated by the results obtained by TEM/STEM (Figure 7). Several previous authors reported lower values of magnetization compared to the protocol presented in this research, using methods such as the thermal decomposition method (76 emu/g) [65] and coprecipitation (60 emu/g) [66]. Research reported that the reduction in magnetization was related to a crystalline disorder, i.e., spin inclination, as a consequence of the reduction of the coordination of surface cations or, in some cases, was linked to negative surface effects promoted by a broken exchange between spins in NP with tiny crystallite size [17,67].

Regarding the method of green synthesis of FeO NPs, previous researchers also reported various values for magnetization in SPIONs, with values lower than those obtained in this investigation, such as 23 emu/g [68], 5.35 emu/g [69], 7.78 emu/g [70], 11 emu/g [71], 0.015 emu/g [72], and 1.57 emu/g [73].

**Figure 9:**
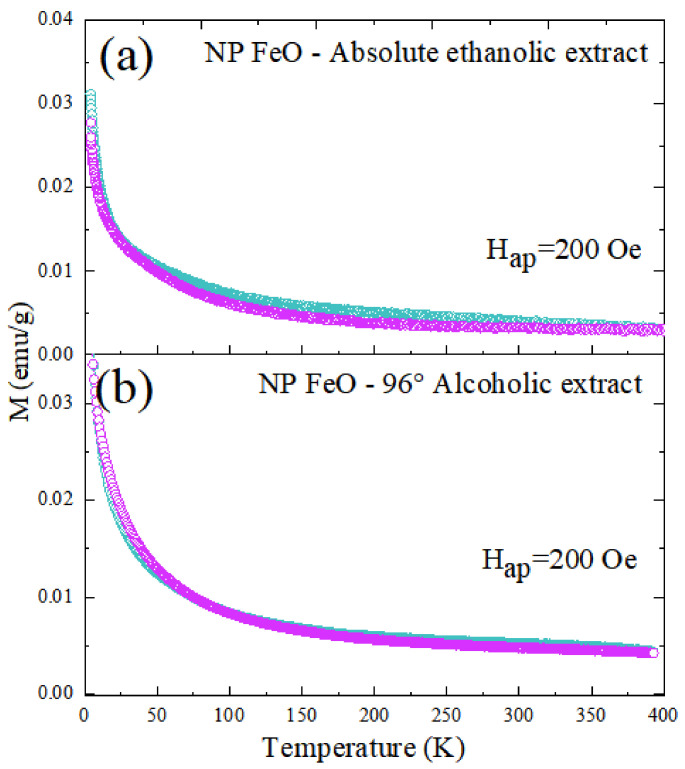
Graph of magnetization as a function of temperature for low fields, with the ZFC and WFC protocols. (**a**) NP FeO Absolute ethanolic extract, (**b**) NP FeO 96% Alcoholic extract.

Figure 9 shows the results of the magnetization response of both of the nanoparticulate colloids under study as a function of the temperature variation. The increase in temperature generated the reduction of the magnetization values for both the measurements of zero field cool (ZFC, orange) and WFC (blue). The response of the WFC measure also described a similar behavior. In both cases, there was no evidence of thermal instability.

### 3.7. Zeta Potential

The stability of FeO NPs colloids is closely related to their surface charge [74]. Thus, it was important to characterize the aforementioned characteristic from electrophoretic mobility. For this characterization, KCl was used at a concentration of 1 mM at pH 6.5. The results of the characterization by Zeta potential showed, for FeO NPs synthesized using *E. globulus* extract in the absolute ethanol solvent, an average of 26.69 ± 2.83 mV and electrophoretic mobility 2.09 ± 0.22 (μ/S/V/cm). For the colloid FeO NPs obtained with the 96% alcohol solvent, an average value of 23.58 ± 2.51 mV and electrophoretic mobility of 1.84 ± 0.2 (μ/S/V/cm) were obtained, showing good colloidal stability. This result reinforced the result evaluated by spectrophotometry (Figure 3). It is important to highlight that colloids with high Z potential (−/+) values are electrically stable, whereas colloids with low Zeta potential (−/+) tend to coagulate [75]. The values obtained indicated minimal or no presence of functional groups and deprotonated biomolecules of the *E. globulus* extract in the colloid, which implied nanoparticles without subsequent reactions. The result obtained in this investigation was comparable to other investigations related to green synthesis, using other types of organic extracts [76,77].

## 4. Discussion

The green synthesis protocols for metallic nanoparticles are mediated by the use of organic extracts, and these in turn are mediated by the presence of metabolites that act as reducing agents of some metallic salt. However, to achieve a complete process of metal precursor reduction, it is necessary to have a high concentration of metabolites. For this, it is important to consider the influence of the type of solvent used for extraction. 

This research provided information, for the first time, on the influence of the use of two types of solvents: the alcohol 96% solvent and the absolute ethanol solvent (99.9%). Extracts in these types of solvents were used in subsequent processes, having a higher alcohol content, On the other hand, aqueous extracts or extracts with a low percentage of alcohol tended to oxidize quickly, which complicated the process.

The diversity of characterizations that were made with respect to the samples under study allowed the consolidation of important information. Thus, the FeO NPs obtained using the extract in the 96% alcohol solvent showed different, but important, characteristics with respect to the colloid obtained using the extract in the absolute ethanol solvent. This difference is specifically linked to colloidal stability, which was initially monitored by UV-vis spectrophotometry and reinforced with evaluation by Zeta potential, where nanoparticle size showed an invariance with respect to the SPR peak, with minimal variation in absorbance.

Both colloidal samples presented spherical morphology. However, the nanoparticles obtained with the absolute ethanol extract showed the presence of organic traces from *E. globulus*, very possibly related to the fact that high alcohol contents allow the extraction of other types of functional groups that do not contribute to reducing activity, remaining in the colloid as traces and bringing variability in the other optical and magnetic properties of the sample. The results of the magnetization measurement showed the presence of SPIONs (superparamagnetic FeO NPs), and in turn it was observed that their properties are defined by the relationships between sizes, surfaces, and crystalline structures, and by an important relationship regarding the magnetic moment, where a lower value is produced due to a possible modification of the three-dimensional frame, evidenced as different magnetization values (emu/g) [78]. The nanoparticulate material obtained was characterized by reaching a state of saturation due to its superparamagnetic nature and consequent response to magnetic fields without delay, which makes it very applicable in regard to environmental remediation issues. In addition, it has excellent Langevin behavior, related to the ability to act in the face of external magnetic fields without maintaining residual magnetism when the same field is eliminated, which implies broadening the applicability in both magnetic resonance imaging and cell separation [79,80,81].

Regarding the mechanism of metal salt reduction by *E. globulus* extracts, the FT-IR characterization suggested that the C=C and C=O groups act as reducing agents, due to the presence of terpenoids and flavonoids.

This research also evaluated the application of the nanostructured material in the removal of heavy metals present in agricultural soil. Only the FeO NPs sample obtained by synthesis using an extract in the 96% alcohol solvent was applied, due to its high monodispersity, smaller size, and colloidal stability, in addition to its better magnetic properties.

The nanoparticles that are most useful for soil remediation are zero valent iron, titanium dioxide (TiO_2_), zinc oxide (ZnO), and multi-walled carbon nanotubes. This is due to their excellent ability to immobilize or adsorb metal ions [82]. Zero valent iron nanoparticles are the most studied for soil remediation, due to their size, large surface area, high reactivity, and reduction capacity. On the other hand, one of the factors that affects the speed of the reaction is the size of the particle [83].

The results obtained from the characterization of the FeO NPs were fundamental in identifying the schematic model linked to the removal of metals. According to XRD analysis, two phases of FeO were identified: magnetite (Fe_3_O_4_) and slight contributions of maghemite (γ-Fe_2_O_3_). The presence of oxide in both phases (magnetite and maghemite) provided the active sites for the adsorption of metals. Additionally, maghemite (γ-Fe_2_O_3_) presented a schematic model with reducing power, confirming the capacity of the obtained NPs in the green route. Previous research indicated that the amount of active sites on the surface is due to the organic functional groups derived from the extract of eucalyptus leaves [52]. This conclusion was reaffirmed in the FT-IR analysis carried out in characterizing the NPs obtained in the green route.

Previous research showed the relationship between the available active sites of the adsorbent and the total amount of adsorbates [84]. The results obtained after 30 min of contact by atomic flame absorption (Table 1) indicated a variance in the adsorption capacity of colloids applied in different concentrations (M1 = 5 mL, M2 = 10 mL, and M3 = 15 mL) for the removal of Cr, since the concentration of the metal (adsorbate) decrease in direct proportion to the mL of NPs (adsorbent) applied.

Table 1 shows the results obtained by atomic absorption. The initial concentration of hexavalent chromium (Cr-VI) was 204.43 ppm, a value that, when compared to the Environmental Quality Standards [85] provided by the Peruvian government for agricultural land, is very high (the maximum allowed concentration is 0.4 ppm). The percentage of chromium reduction by applying the FeO NPs colloids after 30 min was achieved by 100%. The reduction of this metal through the application of NPs resulted from reducing Cr (VI) to Cr (III), where the transfer of electrons took place and Cr (VI) was then reduced to Cr (III) with the oxidation of FeO to Fe (II) and Fe (III), yielding the general chemical Equation (2) [86].

HCrO_4_^−^_(aq)_ + 3Fe^2+^_(aq)_ + 7H^+^_(aq)_ → 3Fe^3+^_(aq)_ + Cr^3+^_(aq)_ + 4H_2_O (2)

On the other hand, the adsorption capacity of Cr (VI) depends on the pH, where the maximum adsorption occurs with pH 2–6. This is due to the dissociation of surface functional groups of the FeO NPs and the speciation of the Cr (VI) ions in the aqueous phase, which are directly influenced by the pH of the solution. In this research, *E. globulus* nanoparticles were obtained with pH 3. This finding was consistent with the research consulted, which indicated that when the pH increases, the adsorption of Cr (VI) decreases, since it would imply a higher concentration of OH^−^ ions present in the dominant formation of chromium, H_2_Cr_4_^−^. This increases the electrostatic repulsion between the adsorbent and the form of the Cr (VI) anion dominant. In addition, adsorption tended to be higher when NPs had a longer contact time with the sample [57,84,86].

On the other hand, the initial concentration of cadmium (Cd) was 0.251 ppm. When compared with the Environmental Quality Standards (ECA), this figure is within the allowed value (1.4 ppm). However, with the application of FeO NPs colloids at different concentrations, with a contact time of 30 min, the removal of 100% of the total Cd was achieved. Previous research indicated that redox reactions dominated the transformation of cadmium from unstable fractions to more stable fractions; in addition, cadmium is immobilized by the combination of adsorption and/or precipitation by the iron oxides formed [56], which confirms the adsorption capacity of the phases found of NP Fe O obtained (γ-Fe_2_O_3_ and Fe_3_O_4_).

Recent studies indicated desorption by soil particles and adsorption on the surface of the nanoparticles in the green route, as they present active binding sites on their surfaces (Equation (3)) [87]. This reaffirms the potential for 100% Cd removal by NP Fe in its two phases.

FeO^−^ + Cd^2+^ → FeOCd^+^ (3)

A study revealed that adsorption reached equilibrium within 30 min [88], a finding consistent with the results obtained in the present investigation, since 100% cadmium adsorption was obtained in the same time. This indicated that Cd adsorption by NPs is primarily chemical adsorption.

Another metal present in the soil sample was lead (Pb), a very common element in the mining industry, with high content in water tributaries that is finally reflected in the soil. Table 1 shows an initial concentration of 497.26 ppm of lead, a value well above what is allowed (70 ppm). As with the previous samples, the same volumes of FeO NPs colloids were used.

Previous research tested the variation of the reducing agent’s influence on the recovery capacity of Pb ions, since an increase in metal concentration occurs when the amount of biomass is increased. This is attributed to the formation of aggregates, due to the electrostatic interactions of the biosorbent that decrease the efficient surface area to allow biosoation [89]. This process explains the stability and increase of Pb concentrations that were found in the present investigation, since only the same concentration of biomaterial was used and there were no variations.

Contact time is also an important factor for the metal sorption capacity of Pb. In this investigation, there was a contact time of only 30 min, which explains the lack of removal. According to previous studies, the ability to remove Pb gradually increases with increasing contact time, from 30 to 60 min [90].

## 5. Conclusions

In this research work, FeO nanoparticles were synthesized using *Eucalyptus globulus* extract as an organic reducing agent to evaluate the influence of the type of solvent used (the 96% alcohol or the absolute ethanol solvent). The FT-IR results revealed that the presence of aromatic compounds are directly involved in the precursor reduction process. The nanostructures obtained showed spherical geometry in both cases, with sizes of 2.34 and 4.17 nm for FeO NPs in the absolute ethanol and alcohol 96% solvents, respectively. In addition, the presence of the magnetite and maghemite magnetic phases was confirmed by XRD characterization. Elemental analysis by EDS showed better iron purity when the solvent was used in low concentration alcohol. The magnetic response exhibited, in both cases, a behavior attributable to superparamagnetic materials. The application of FeO NPs obtained with the best synthesis (using the 96% alcohol solvent extract) in soil remediation was successful for metals such as chromium and cadmium. This mechanism was linked to the presence of oxygen in the nanostructure, providing active sites for the metal absorption.

## Figures and Tables

**Table 1:** Quantification of the removal of heavy metals present in agricultural soil through the application of FeO NPs.

	Chrome				Cadmium				Lead			
Sample	Abs	Initial ppm	Final ppm	% Removal	Abs	Initial ppm	Final ppm	% Removal	Abs	Initial ppm	Final ppm	% Removal
M3	−0.079	204.43	−34.01	100%	0	0.251	0	100%	0.15	497.26	438.54	11.8%
M2	−0.269		−114.28		0.005		−0.10		0.17		497.26	0%
M1	−0.294		−124.85		0.003		−0.20		0.27		790.86	0%

## Data Availability

Not applicable.

## Supplementary Materials

The following supporting information can be downloaded online, Figure S1: EELS spectra of O-K edges (**a**) and Fe-L2,3 edges (**b**) acquired from a FeO NP 96% alcoholic extract (red curves) and FeO NP absolute ethanolic extract (blue curves), respectively; Figure S2: Green FeO NP synthesis protocol, using 96% alcoholic and absolute ethanolic extracts of *E. globulus*.
