# Supplementary material for: Green Synthesis of Superparamagnetic Iron Oxide Nanoparticles with Eucalyptus globulus Extract and Their Application in the Removal of Heavy Metals from Agricultural Soil
Source: Molecules. 2022 Feb 17;27(4):1367. doi: 10.3390/molecules27041367 (PMC8880537; doi:10.3390/molecules27041367)
Supplement: Supplementary file 1 [file molecules-27-01367-s001.zip › molecules-1571311-supplementary.pdf]

Supporting Information

Characterization by Electron Energy Loss Spectroscopy (EELS)

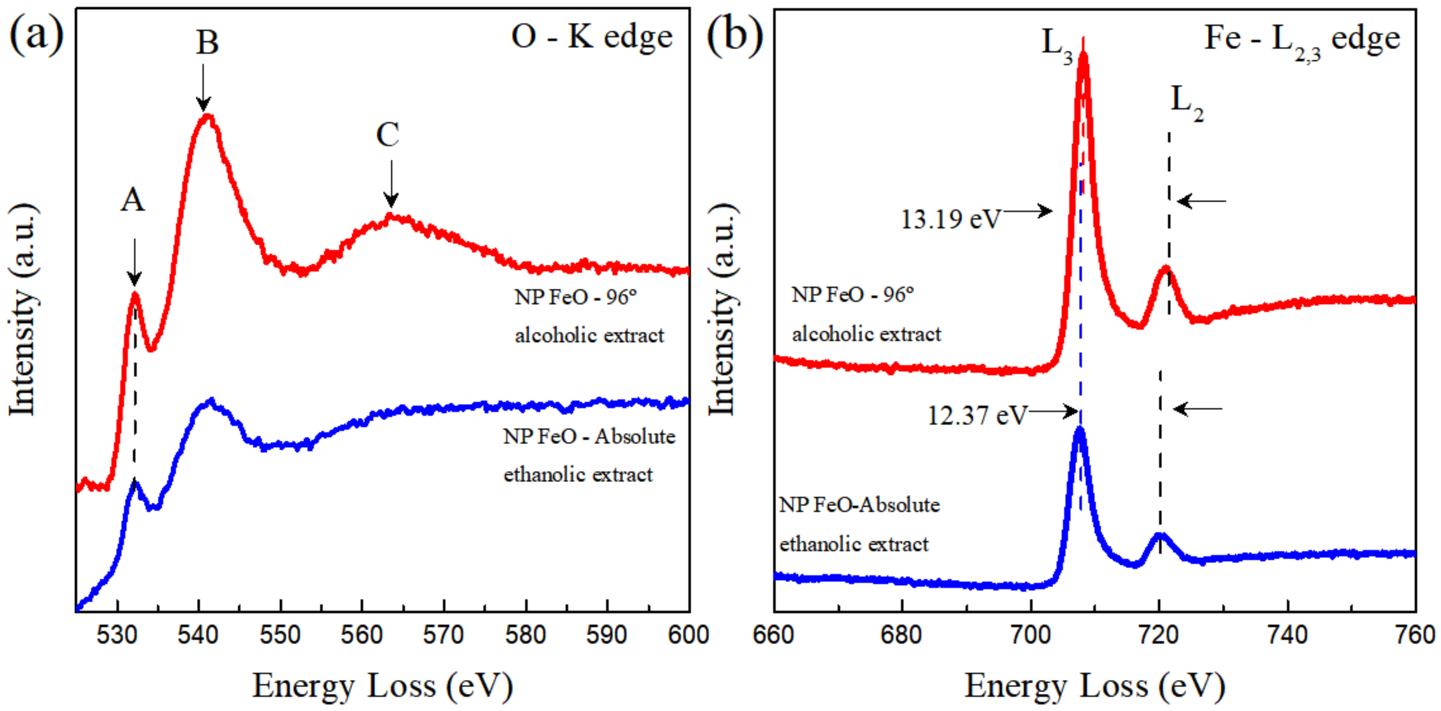

Figure S1. EELS spectra of O-K edges (**a**) and Fe-$L_{2,3}$ edges (**b**) acquired from a FeO NP 96% alcoholic extract (red curves) and FeO NP absolute ethanolic extract (blue curves), respectively.

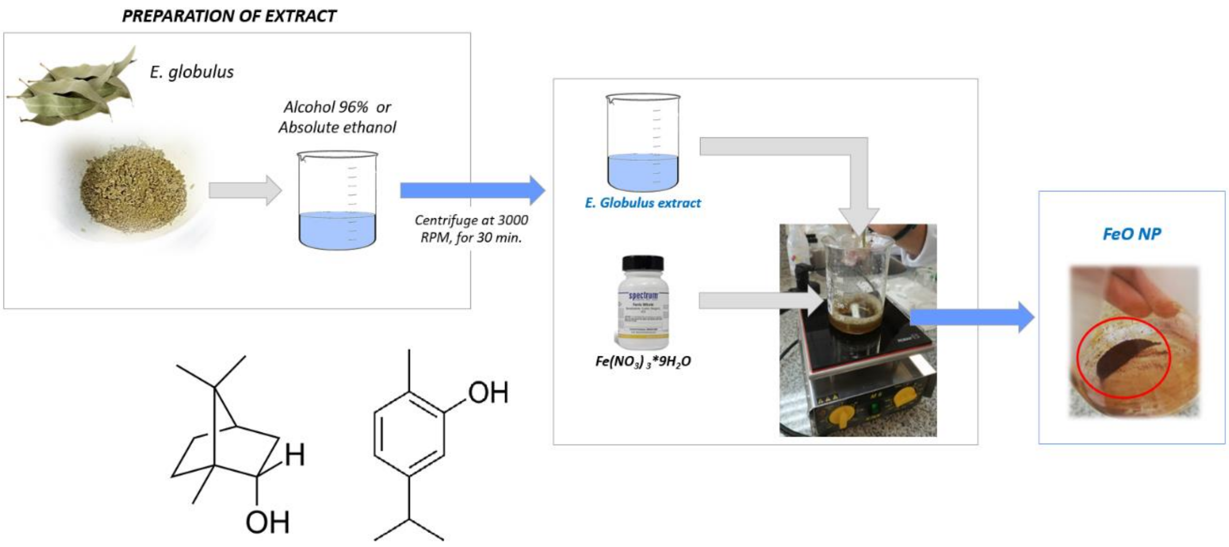

Figure S2. Green FeO NP synthesis protocol, using 96% alcoholic and absolute ethanolic extracts of E. globulus
